# Testicular Torsion in the Left Inguinal Canal in a Patient with Inguinal Hernia: A Difficult Case to Diagnose

**DOI:** 10.4274/MIRT.017978

**Published:** 2011-12-01

**Authors:** Alihan Erdoğan, Emel Ceylan Günay, Gökhan Gündoğdu, Dincer Avlan

**Affiliations:** 1 Mersin University Faculty of Medicine, Department of Nuclear Medicine, Mersin, Turkey; 2 Mersin University Faculty of Medicine, Department of Pediatric Surgery, Mersin Turkey

**Keywords:** Spermatic cord torsion, Radionuclide imaging, Cryptorchidism, inguinal hernia

## Abstract

A 8 month old boy suffering from inconsolable cry and tenderness presented to our hospital. Ten hours had passed from the onset of his symptoms. Physical examination showed a tender mass on the left groin. Patient was evaluated with Doppler ultrasound and Technetium-99m pertechnetate testicular scintigraphy Differential diagnosis of torsion and inflammatory disease could not be made by adjunctive tests. The patient underwent surgery based on clinical findings and necrotic inguinal left gonad was shown. In this study, we discussed the scintigraphic pattern in a patient with torsion in undescended inguinal testicle

**Conflict of interest:**None declared.

## INTRODUCTION

Testicular torsion is a well known urologic emergency that needs to be diagnosed and treated rapidly for the salvage of testis (1). Sometimes it could be difficult to differentiate this event from inflammatory diseases of the testes. Differential diagnosis could even be more difficult in undescended testis which is an uncommon cause of inguinal swelling in children (2,3). We report here a case of torsion within the left inguinal canal, and discuss the diagnostic evaluation.

## CASE REPORT

An 8-month-old infant who has discomfort and inconsolable cry for ten hours was admitted to our hospital. Physical examination showed a tender mass on the left inguinal region. Previously, the patient had a diagnosis of left inguinal hernia and undescended testis.

The left testis was found in the inguinal canal, 16x12 mm in diameter by Doppler ultrasound. This testis had significantly heterogeneous parenchyma compared to contralateral testis. Peritesticular tissues in this region were oedemateous and increased vascularity was shown at the left epididymal localization. There was no abnormality in the right testis. Differential diagnosis could not be made.

**Figure 1:**
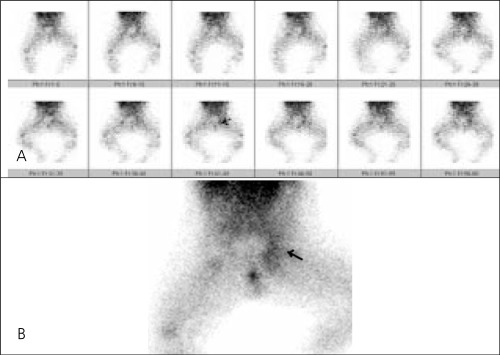
Dynamic images (a) and static image (b) in Tc-99m pertechnetatetesticular scintigraphy showing diffusely increased activity in the left inguinalregion (arrow)

Testicular scintigraphy was performed after the intravenous injection of 3 mCi Tecnetium-99m (Tc-99m) pertechnetate. Flow images and blood pool images of 500.000 counts were obtained in anterior view in a supine position with a large field of view gamma camera using low-energy high resolution parallel hole collimator. The left inguinal mass showed diffusely increased activity (Figure 1a-b). There was no significant photopenic area leading to diagnosis of torsion. Scintigraphic findings were normal for the right scrotum.

Since torsion was more likely suspected in the clinical evaluation, surgical exploration of the inguinal region has been decided. At surgery, the testis was seen as necrotic and twisted 720 degrees intravaginally. After detorsion and heating, there was no recovery for the testis and orchiectomy was performed. The patient's post operative course was uneventful.

## LITERATURE REVIEW AND DISCUSSION

It is important to differentiate the diagnosis of testicular torsion from the inflammatory diseases such as epididymitis or orchitis in acute scrotum. Delayed diagnosis of testicular torsion may result in necrosis or atrophy. On the other hand, non-surgical clinical events could lead to unnecessary surgery. Although the normal anatomic localization of testes give the chance of easy examination, sometimes it might be difficult to differentiate the diagnosis of acute scrotum, especially in early diagnosis (4). It could even be more difficult when testes are located out of the scrotum. Differential diagnosis also includes incarcerated inguinal hernia, ileus, acute appendicitis, trauma and abdominal or testiculartumors (5,6). Although undescended testes are mostly localized in the inguinal canal, torsion of the inguinal testicle is a rare situation than other localizations of cryptorchid testes. It has been proposed that hernia and relatively short cord seen in inguinal testis makes inguinal torsion relatively more difficult (7,8). However, the lack of anatomic fixation of the testes in the scrotum and the possibility of spasmodic contractions of the cremaster muscle may result in torsion of the undescended testis.

Radionuclide imaging and Doppler ultrasonography can be performed for differential diagnosis of testicular torsion. The sensitivity of the radionuclide imaging which has been used for this cause for over 30 years is reported within 90 to 100% (1,9,10). Scintigraphic findings of testicular torsion could show some variations according to the time left from the onset of acute event. In early diagnosis, flow images usually seem symmetrical but sometimes proximal spermatic vessels show some activity. A typical photopenic area is seen with no encircling increased activity in soft tissue images. In contrast, the inflammatory diseases show increased activity both in flow and soft tissue phase without a photopenic area (11,12). Delayed torsion shows a photopenic area surrounded by a rim of increased activity on tissue phase called 'missed testicular torsion'. It has been reported that if torsion is delayed enough, flow images can also show the increased activity encircling the involved testicle (13). In our case, there was no photopenic area to indicate testicular torsion. Moreover, we also observed diffusely increased activity in the inguinal region on the affected side. Scintigraphic images could easily be interpreted as inflammatory diseases, considering the time of the event and the increased activity on tissue phase. We assume that the increased activity in radionuclide study was the result of the oedema and the inflammation of the surrounding tissues which attenuated the pathological sign of testicle with the torsion. The inflamed soft tissue was so thick that neither scintigraphy nor Doppler ultrasonography could be able to make accurate diagnosis. The patient had gone to surgical exploration depending on clinical suspicion. Final diagnosis was made by surgical findings and pathological results.

There are a few published cases with torsion of undescended inguinal testis diagnosed with testicular scintigraphy based on photopenic area (6,14). Scintigraphy can confirm the clinically suspected diagnosis of torsion. However, nuclear physician should be very careful while interpreting increased activity in the inguinal region. Any photopenic region belonging to undescended twisted testicle might remain in the shade of the inflamed surrounding tissue which is probably thicker than normal scrotum.

Although rare, torsion of undescended testis should be considered in a child presenting with abdominal groin pain and empty ipsilateral scrotum. Nuclear physician should be very careful before eliminating the diagnosis of torsion.
